# High-Resolution Ultrasound to Quantify Sub-Surface Wrinkles in a Woven CFRP Laminate

**DOI:** 10.3390/ma17092002

**Published:** 2024-04-25

**Authors:** Md Admay Amif, David A. Jack

**Affiliations:** Department of Mechanical Engineering, Baylor University, Waco, TX 76706, USA; admay_amif1@baylor.edu

**Keywords:** woven fiber carbon composite, out-of-plane wrinkle, conventional ultrasound, A-scan, peak tracking

## Abstract

Carbon fiber reinforced polymer (CFRP) composites are popular materials in the aerospace and automotive industries because of their low weight, high strength, and corrosion resistance. However, wrinkles or geometric distortions in the composite layers significantly reduce their mechanical performance and structural integrity. This paper presents a method for non-destructively extracting the three-dimensional geometry, lamina by lamina, of a laminated composite. A method is introduced for fabricating consistent out-of-plane wrinkled CFRP laminate panels, simulating the in-service wrinkle observed in industries that utilize thick structure composites such as the vertical lift or wind power industries. The individual lamina geometries are extracted from the fabricated coupon with an embedded wrinkle from captured ultrasonic waveforms generated from single-element conventional ultrasonic (UT) scan data. From the extracted waveforms, a method is presented to characterize the wrinkle features within each individual lamina, specifically the spatially varying wrinkle height and intensity for the wrinkle. Parts were fabricated with visibly undetectable wrinkles using a wet layup process and a hot press for curing. Scans were performed in a conventional immersion tank scanning system, and the scan data were analyzed for wrinkle detection and characterization. Extraction of the layers was performed based on tracking the voltage peaks from A-scans in the time domain. Spatial Gaussian averaging was performed to smooth the A-scans, from which the surfaces were extracted for each individual lamina. The extracted winkle surface aligned with the anticipated wrinkle geometry, and a single parameter for quantification of the wrinkle intensity for each lamina is presented.

## 1. Introduction

Carbon fiber reinforced polymer (CFRP) composites are lightweight, strong materials used in various industries. They offer a significantly higher strength-to-weight ratio in comparison to metals. Industries such as aerospace, automotive, construction, and machinery benefit from CFRP composites [1,2]. Numerous studies have investigated aspects of carbon fiber reinforced polymer (CFRP) composites, including layup sequences [3]; ply orientations [4,5]; bond line thickness [6]; and the exploration of defects such as foreign object debris (FOD) [7], barely visible impact damage (BVID) [8], out-of-plane wrinkles [9], porosity measurement [10], interlaminar delamination [11], etc. In this study, we present a method for characterizing out-of-plane wrinkles from UT scanning data in carbon fiber reinforced polymer (CFRP) composites.

The reasons behind a wrinkle being present within a laminated composite are induced during manufacturing from a variety of sources, such as micro-scale deformations, non-uniform pressure distributions, poor co-bonding between lamina, thermal coefficient (CTE) mismatch, gaps, overlaps, etc. [12]. Hallander et al. [13] observed that the lay-up sequence significantly influenced out-of-plane wrinkling in UD prepreg laminates, even when the other variables inducing wrinkles are mitigated. Hallander et al. showed that even for quasi-isotropic, multilayer UD prepreg structures on a double curved geometry, out-of-plane wrinkling can occur.

**Figure 1:**
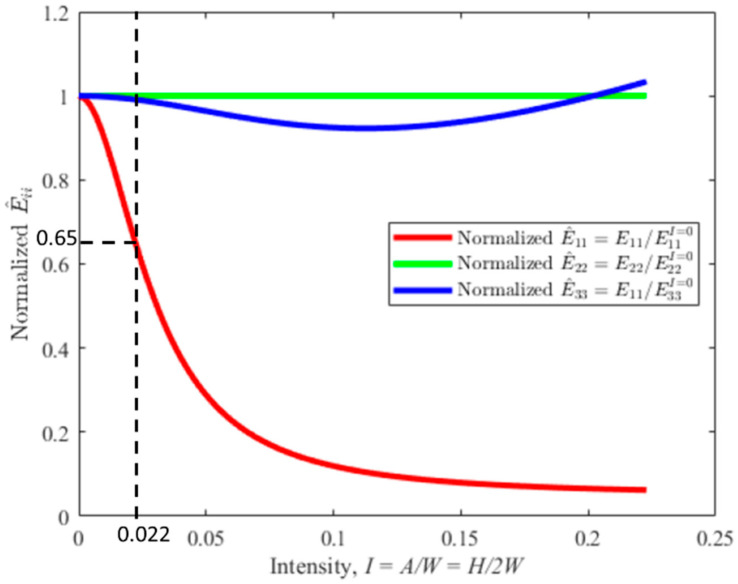
Effect of wrinkle intensity on normalized stiffness values along each axis using the Hsiao–Daniel model.

The structural capacity of a laminated part is compromised due to the presence of a wrinkle. For example, Xie et al. [14] performed a numerical study to relate the maximum wrinkle angle and cross-sectional area with knock-down in failure stress, which can be as large as a 50% reduction in failure stress. Similarly, Hsiao and Daniel [15] provided an analytical model to predict the elastic properties of a composite part. An example is provided in Figure 1 of the change in material stiffness as a function of the wrinkle intensity I, defined as the ratio of the wrinkle height H divided by twice the winkle width $\left(2W\right)$, specifically I=H/2W. This figure was plot using a custom in-house code of the published Hsiao and Daniel model. Note that the point of 0.022 is highlighted as this is the actual wrinkle intensity of the part studied in the results section of the present paper. It is important to note that a wrinkle intensity of 0.022 is almost imperceptible to the trained eye, whereas it reduces the stiffness along the fiber, $E_{11}$ by nearly 35%. Detecting and quantifying fiber waviness is crucial for maintaining quality during manufacturing, and it is necessary to have an inspection method for CFRP materials [16].

It is desirable to develop a non-destructive testing (NDT) approach to quantify embedded wrinkles, to prevent compromised parts from finding implementation in service (see, e.g., [9,17]). There are a variety of NDT methods commonly employed when studying structures, such as electromagnetic testing (ET), ultrasonic testing (UT), thermographic testing (TT), radiographic testing (RT), computed tomography (CT), shearography, etc., and many have found use in inspections of CFRP composites (see [18,19]). UT and X-ray CT are the two primary approaches for inspecting laminated polymer matrix composites, due to their accuracy, with the remaining aforementioned techniques finding limited use and acceptance. Due to the expense, time, limited component sizes, and complexity of CT technology, UT systems have a distinct advantage, especially with recent advances in digitizing and higher frequency waveforms [9,20]. Ultrasound has shown tremendous success when it comes to detecting and quantifying different embedded features in composite laminate parts, but most work is carried out using manual methods requiring highly trained operators to interpret the captured waveforms.

Sandhu et al. [21] presented an analytical approach to extract bulk wrinkle characteristics, i.e., wavelength and amplitude from low resolution B scans, with their algorithm converting rectangular scan coordinates into part coordinates for analysis of a curved composite part. The information generated by Sandhu et al. provides general wrinkle characteristics but does not yield quantifiable information for individual lamina. Larranaga-Valsero et al. [22] also detected wrinkle characteristics of height, severity, and maximum angle of a hybrid composite part with unidirectional carbon and woven glass fiber using the FMC/TFM method of phased array. They analyzed the instantaneous amplitude and phase analytically at the first and second resonance frequencies for optimum frequency and then simulated at the given frequency, and from the analyzed data, they were able to visually interpret the results to identify the wrinkle. Zhang et al. [23,24] observed the trend in signal intensity where inter-ply reflection kept increasing with the increase in frequency, and wrinkle defect reflection showed the opposite trend in a rich resin thick composite. They analyzed B-scan images of side drilled holes (SDH) and rich resin areas by filtering the center frequency from 2.5 MHz to 6 MHz and compared them with the simulated result. From the analyzed data, Zhang et al. were able to interpret plotted scan data to identify the wrinkle. The present authors have been unable to identify in the literature a method that automates the extraction, on a lamina-by-lamina basis in three dimensions, of the wrinkle features of each individual lamina.

**Figure 2:**
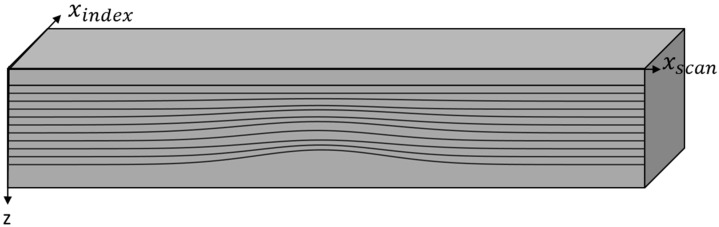
Coordinate system used for the scan analysis.

In this paper, an algorithm is presented to take conventional ultrasonic data, extract the geometric position in 3D-space of each of the individual layers of a carbon fiber laminated composite, and then provide a bulk characterization of the wrinkle height and intensity for each layer. The presented method is not necessarily limited to carbon fiber filled systems, and future studies may consider alternative material systems. Figure 2 represents a wrinkle along with the coordinate system used for the results, where the $x_{scan}$, $x_{index}$, and z axes represent the scan direction, index direction, and depth from the top of the surface, respectively. The concern is that an embedded wrinkle would not be visible from the surface, thus the need for a non-destructive approach to capture the wrinkle. An additional uniqueness of this work is the application to woven lamina, whereas existing works addressed wrinkles in unidirectional laminates. In addition, the spatial variation in the wrinkle along with a layer-by-layer characterization is also unique to this work. The present study focuses on wrinkles up to the 14th layer of a laminated composite. Future studies are needed to investigate the limitations of the proposed method as composite structures become thicker. The authors have reason to believe that thicker laminates could be investigated, contingent upon there being a sufficient signal-to-noise ratio of the captured reflection wave as one penetrates deeper into the laminate.

## 2. Manufacturing Method

There are several ways of fabricating a woven CFRP laminate. Laminated composites have found widespread use in the aerospace industry. In the present research, we selected a wet layup fabrication with subsequent curing utilizing a hot press. This approach allowed us to tune the wrinkle characteristics. The wet layup process continues to find its way into the manufacture of large components with complex shapes, but the methodology presented is not limited to this manufacturing process. Based on several internal studies, this approach yielded acceptable manufactured wrinkles that effectively simulated wrinkles from several proprietary parts we observed.

### 2.1. Wet Layup

**Figure 3:**
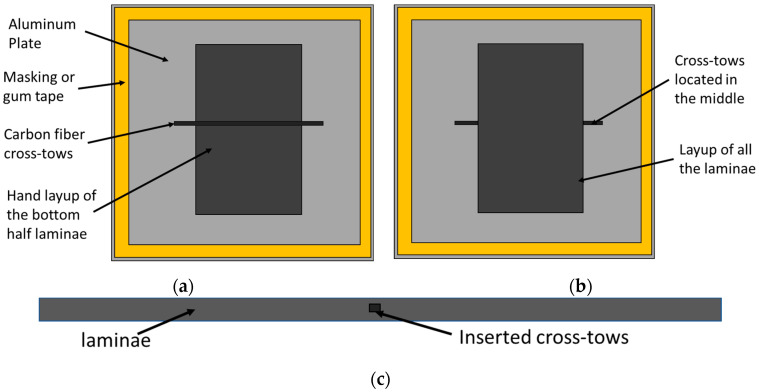
Schematic diagram of the fabrication process, (**a**) indicating the cross tows placed between the center lamina, (**b**) the overall layup before curing with all lamina, and (**c**) a side view of the carbon fiber laminate layup showing the embedded tows.

The wet layup procedure is a hand layup technique where resin is applied to dry fiber with a brush. In this study, dry fibers were placed one after another, and resin was manually wiped onto the lamina surface before placing the next lamina. Two aluminum plates were prepared using a mold release agent, one for use as a tool on which the wet layup was performed, and another to put on top of the laid-up laminae. Then, 3K plain weave carbon fibers from ACP Composites were used for fabricating the laminate. The resin system was a Pro-Set Infusion Epoxy Resin 114 (INF-114) and a Pro-Set Infusion Epoxy 211 Hardener (INF-211) (Pro-Set, Inc., Bay City, Michigan) with a prescribed mix ratio of resin and hardener of 3.65 to 1. A FlakTek SpeedMixer (FlackTek Manufacturing Inc., Louisville, CO, USA) was used to remove bubbles and adequately mix the resin and hardener. The mixer was programmed to spin in a vacuum at 800 rpm for 30 s and then at 1500 rpm for 270 s. After laying up about half of the rectangular carbon fiber laminas, in the present study, 14 lamina multiple 3K tows were placed across the layup, as shown in Figure 3a. Then, the remaining laminae were placed on the layup, repeating the fabric and resin application process, as shown in Figure 3b.

The cure cycle for the selected resin system calls for the part to be held under pressure for 8 h, and then placed in an elevated temperature environment to complete the curing process. In the present study, the part was kept at room temperature for 6 of the 8 h gelation time before moving the layup to the pressure step, similar to the approach suggested by a previous researcher in Minnie [9].

### 2.2. Curing

The recommended curing from the manufacturer requires 8 h at room temperature followed by 8 h at 82 °C. After 6 h of gelation, the laminate was kept in the hot press for 10 h. During the elevated temperature portions of the study, along with the final 2 h of the room temperature cure, a holding pressure of 276 kPa (40 psi) was applied. A programmable hot press, a Carver Auto Four/1512-PL (Carver Inc., Wabash, IN, USA), was used for curing the laminate.

## 3. Analysis Methods

### 3.1. Ultrasonic Data Collection

**Figure 4:**
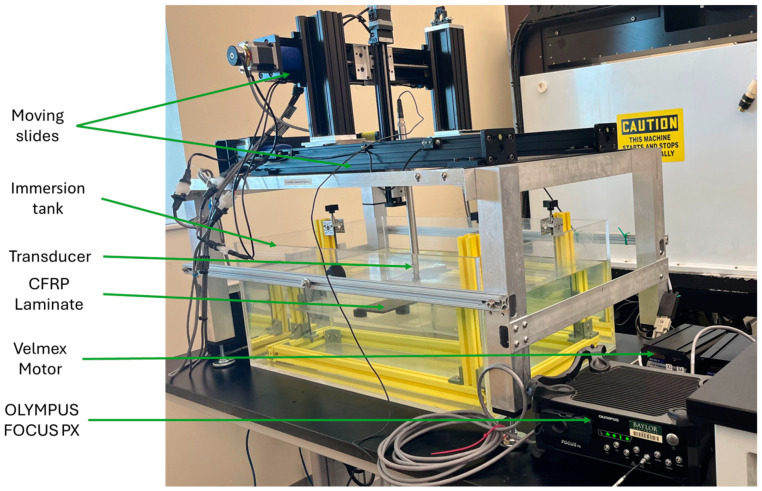
Immersion tank system utilized to perform scans in the present research.

**Figure 5:**
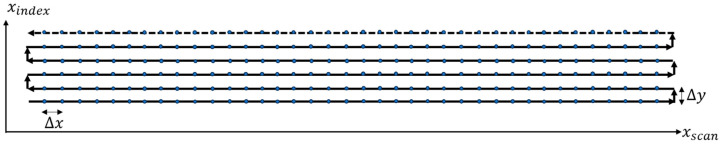
Typical raster pattern used to scan the region of interest.

The ultrasonic scan system used for this study was a custom immersion tank scanning system with a single spherically focused transducer. The advantages of this type of scan include a uniformity of acoustic coupling that reduces sensitivity variations, a reduction in the scan time due to automation, and the focused immersion transducer increasing the sensitivity to small reflectors [25]. The UT scan was performed in a water tank, as shown in Figure 4, using pulse-echo scanning with access to only a single side of the laminate (see e.g., [20,25]). Based on several internal studies, a single-element 37.5 mm spherically focused probe with a 7.5 MHz frequency was found appropriate over a wide range of laminate thicknesses. The transducer was excited to 190 V, the largest value allowed by the Olympus Focus PX (Evident, Center Valley, Lehigh County, PA, USA) digitizer utilized, with a square wave pulse width of 65 ns. Two Velmex Bi-slides with a spatial resolution of 0.0025 mm, as shown in Figure 4, were used to move the transducer along the scan axis and then in the index direction following the raster pattern, as shown in Figure 5, to scan the region of interest (ROI) of the laminated part. A step size of Δx=Δy=0.2 mm was used in the present study. It is noted that scans typically took 10 min in the present investigation. The scan time was inversely proportional to the scan resolution; thus, a doubling of the index size, resulted in a nearly 50% reduction in the scan time. Moreover, a subject which the authors are currently pursuing is to implement a synthetic raster using phased array, often reducing the scan time by an order of magnitude.

For this study, the scan direction generally traversed transverse to the embedded cross tows, and the index direction generally aligned with the embedded cross tows. A-scans and B-scans were produced and analyzed to study the sub-surface wrinkles. An A-scan is a one-dimensional scan where the scan-echo amplitude is plotted as a function of time, and a B-scan displays a series of A-scan readings that originate in a single run along a single axis [25].

### 3.2. Extraction of Laminated Layers

In this study, an algorithm was constructed to extract the three-dimensional lamina position from within the laminated composite part. The algorithm allowed the quantification of the bulk wrinkle parameters, specifically the wrinkle height and wrinkle intensity, the results of which are presented in Section 4.

**Figure 6:**
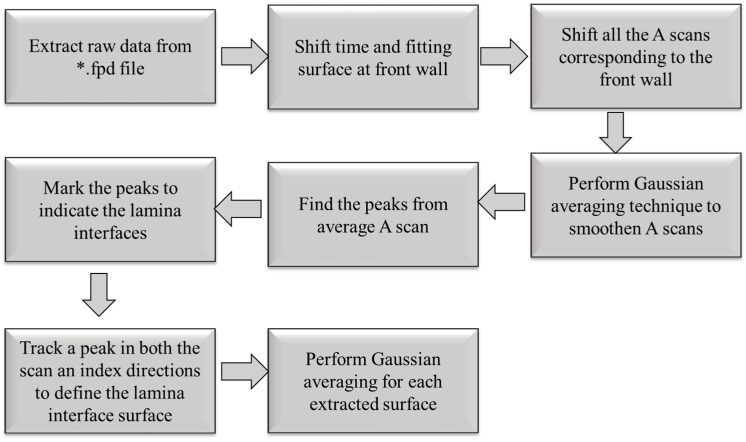
Flowchart of the layer extraction method, implemented in the MATLAB environment.

**Figure 7:**
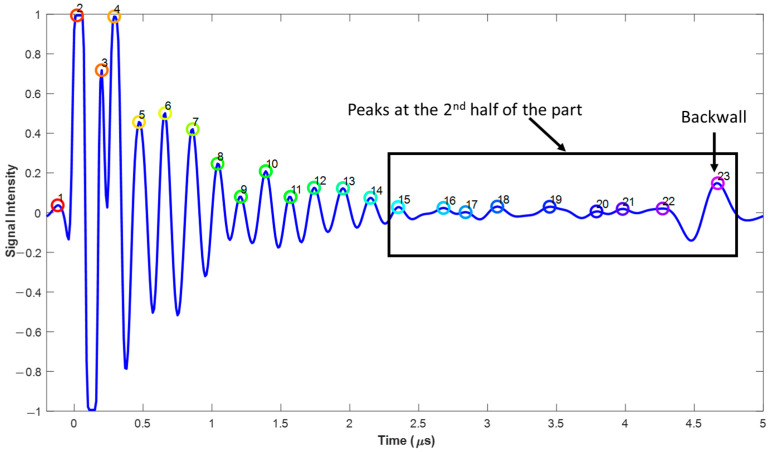
Average A-scan used for lamina interface detection.

**Figure 8:**
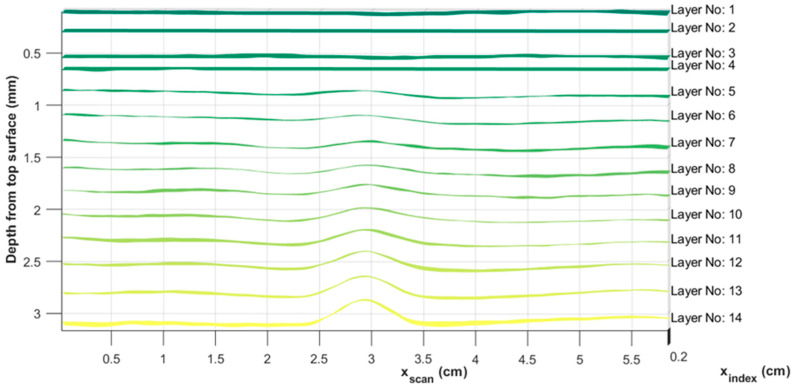
Extracted layer surfaces from the analysis of the first 14 lamina.

Figure 6 represents the algorithm of the overall layer extraction method. Data were initially captured using an Olympus Focus PX (Evident, Center Valley, Lehigh County, PA, USA) digitizer and saved in an Olympus proprietary file format, *.fpd. The raw A-scan data were then read into a MATLAB (version 2022b) script and then shifted in time such that the initial reflection wave from the front of the part was aligned across all A-scans. Next, a spatial Gaussian averaging technique (see [26] for a presentation of this algorithm) was performed to smooth the data in the plane of the laminate for a given depth. Next, the averaged A-scan over a subregion was compiled and the individual peaks in time were extracted from the waveform. The results of a typical peak detection can be seen in Figure 7. Observe that the individual peaks correlated to the interface between lamina, and these peaks were tracked between each of the A-scans within the region of interest, forming a layer-by-layer surface. Each peak indicates an individual depth of the laminated part corresponding to the depth of each individual ply. The individual peaks were then tracked in both the scan an index directions. This tracking resulted in a surface, with the vertical dimension corresponding to the lamina position, given as the time of flight, within the laminate as a function of $\left(x_{index},x_{scan}\right)$. This extracted surface was then smoothed using a Gaussian averaging technique, and the time of flight data were converted to depth data using the effective through thickness speed of sound of the laminate to extract the layers as a function of the individual layer depth, as shown in Figure 8 and plotted in the MATLAB environment. The effective through thickness speed of sound was obtained using the known thickness of the part times two, divided by the time of flight of the signal between the front wall and the back wall. The addition of the two was made as we utilized the pulse-echo mode of inspection.

### 3.3. Scan Data Analysis

Figure 7 represents a typical average A-scan over a sub-region of size x.xx (mm)×y.yy(mm) of the overall 58.4 mm×10 mm scan. This subregion scan was taken far from the wrinkle and was used as the seed A-scan, where each of the corresponding peaks indicate the interface between the laminae. Peaks above a prescribed threshold, in the present study we used 0.02, were identified as being an interface between lamina. This worked effectively for lamina above the inserted tow layer, specifically the interface between the 14th and 15th lamina, but did not capture the interfaces for lamina past the 15th lamina. Specifically, as the synthetic part of 28 lamina had the embedded 3K tows between the 14th and 15th lamina, it did not make sense to analyze any of the lamina deeper than the 14th lamina. A manufactured component would not typically have additional tows causing a wrinkle; thus, it was not reasonable to analyze the lamina beneath the embedded tow layer. Each peak within the locally averaged A-scan was tracked across the surface of the part. It is worth noting that the signal intensity tended to decrease while penetrating the part because of the signal attenuation, with a high intensity at the back side of the part reflecting off the interface between the laminate and the surrounding water medium. The various layers are identified in Figure 7, along with the backwall. As shown in Figure 7, the signal weakened due to signal attenuation.

**Figure 9:**
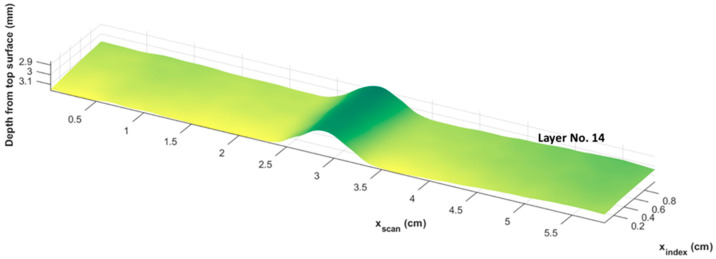
Three-dimensional representation of the 14th lamina of the part showing the subsurface wrinkle.

Figure 8 represents a side view of each of the individual lamina, as extracted from the automated algorithm presented in Figure 6 and implemented in the MATLAB software environment. The ROI was taken as a 58.4 mm × 10 mm area with an overall part thickness of 6.23 mm. Of note is the 14th layer of the part that exhibits the highest wrinkle intensity in Figure 8. The top four layers of the part are almost flat, which is in agreement with the visual inspection that was unable to identify any wrinkles from the part surface. Conversely, the wrinkles in the individual layers can be observed to progressively increase from lamina 5 up to the 14th lamina. The 14th lamina is plotted separately in Figure 9, plotted in the MATLAB environment. Notice that the 3D representation of the 14th lamina is reasonably uniform along the axis of the wrinkle, but there are subtle changes along the projection of the wrinkle peak, a detail expanded upon in the next section and highlighting that the present method can capture the spatial variations in the wrinkle intensity.

## 4. Results

**Figure 10:**
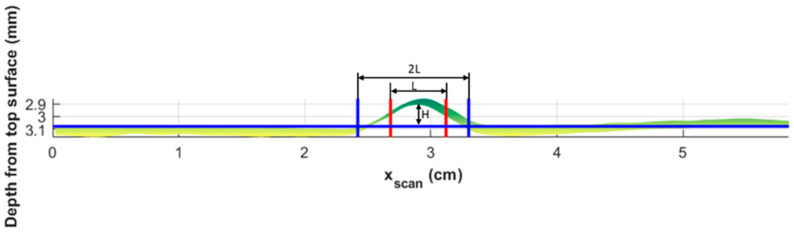
Wrinkle intensity characterization of the 14th extracted lamina.

For this study, two sub-surface wrinkle parameters, wrinkle height and wrinkle intensity, were analyzed. As the extracted surface was three-dimensional, a baseline was created by fitting a linear surface to the lamina well outside of the region containing the wrinkle. The wrinkle height H, such as that shown in Figure 10, was measured for each value of $x_{index}$ for each of the individual scans. The base length of the wrinkle was defined as 2L (see Figure 10), where L is the length between the two points where the layer height is one-half of the wrinkle height. The wrinkle intensity is defined as follows (similarly to that in, [15,23,27]):

(1) $$I=\frac{H}{2L}$$

**Figure 11:**
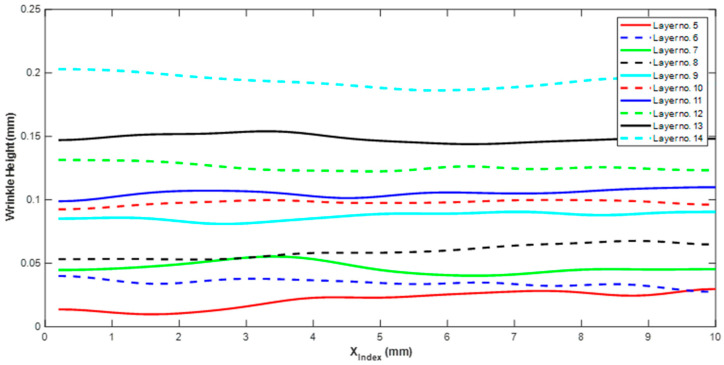
Wrinkle height for each of the individual lamina along the index direction.

**Figure 12:**
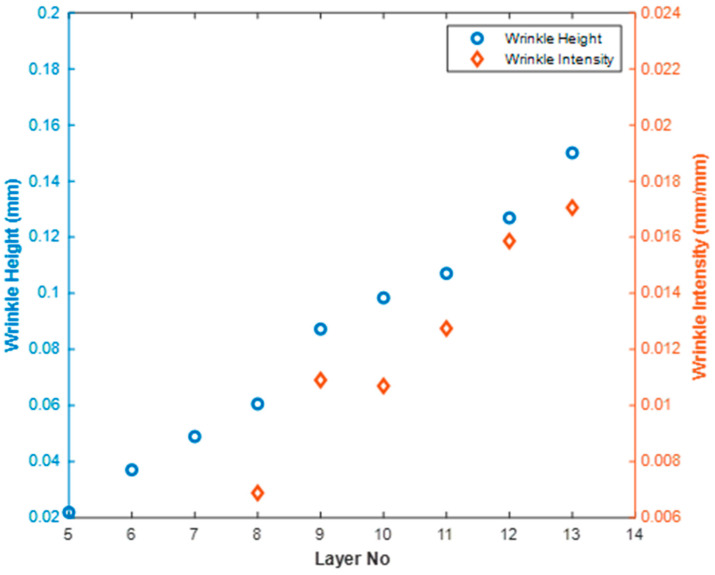
Non-destructively extracted wrinkle height and wrinkle intensity as a function of depth within the investigated laminate.

Figure 11 represents the wrinkle height along $x_{index}$ for each of the 14 laminae. Observe that the height was not constant across the $x_{index}$ direction but it did remain fairly consistent, and an average value is reported to represent the lamina. The wrinkle height generally increased monotonically for each layer as one progressed deeper into the laminate. For example, the wrinkle height was measured as 0.01 mm at layer 5, whereas the maximum wrinkle height was found to be 0.19 mm at layer 14. To report the wrinkle height, a spatial average was computed, and the average wrinkle heights are shown for each layer in Table 1 and Figure 12.

The top four laminae are not reported, as no detectable wrinkle was identified. Although a wrinkle height could be quantified for the 5th through 7th lamina, the wrinkle width was quite difficult to quantify due to uncertainty, and thus the intensity is not reported for these laminae. For lamina 8 through 14, both the height and intensity are reported and presented in Table 1. The wrinkle intensity was measured as a minimum of 0.0069 in the 8th lamina, and the maximum intensity was found to be 0.0221 on the 14th layer, which was the closet layer to the inserted cross-tows in the part. Looking back to Figure 1, this would suggest that the 14th lamina would have a reduced stiffness of 65% that of the outer most lamina, thus the outer lamina would carry more of the load in any structural application.

## 5. Conclusions and Discussion

Wrinkles are a common defective feature in a manufactured CFRP composite part, which can significantly compromise the structural performance of a CFRP. The current literature does not provide examples of the extraction, on a lamina-by-lamina basis and in three dimensions, of the wrinkle feature of each individual lamina.

- The present research presents a method for fabricating a laminated composite with a synthetic wrinkle that can be used for inspection methodology development.
- The present research presents both a methodology and results for the extraction of the wrinkled layer surfaces from ultrasonic data.
- The automated code can extract the spatially varying wrinkle geometry and quantify the wrinkle intensity, height, and width as a function of spatial position, for each individual lamina.

In the present study, a part with 28 lamina that were mirrored about the central axis with embedded tows creating an internal wrinkle was studied. The presented results include the 3D surface of each individual lamina, from which the height of the lamina and the wrinkle intensity could be readily characterized. The results presented included a wrinkle with a height as small as 0.01 mm and a wrinkle intensity of only 0.0069 up to a wrinkle height of 0.19 mm and an intensity of 0.022. Future work needs to include validation of the results from sectioned samples characterized using microscopy and potentially a full X-ray CT inspection of the laminate.

## Figures and Tables

**Table 1:** Wrinkle heights of the individual lamina from the automated non-destructive inspection, where N/A indicates the results are not applicable for analysis.

Layer No.	Wrinkle Height (mm)	Wrinkle Intensity (mm/mm)
1–4	N/A	N/A
5	0.0210	N/A
6	0.0321	N/A
7	0.0435	N/A
8	0.0574	0.0069
9	0.0866	0.0109
10	0.0968	0.0107
11	0.1029	0.0127
12	0.1246	0.0159
13	0.1473	0.0171
14	0.1931	0.0221

## Data Availability

The raw data supporting the conclusions of this article will be made available by the authors for reasonable requests.
